# Contribution of the Potassium Channels K_V_1.3 and K_Ca_3.1 to Smooth Muscle Cell Proliferation in Growing Collateral Arteries

**DOI:** 10.3390/cells9040913

**Published:** 2020-04-08

**Authors:** Manuel Lasch, Amelia Caballero Martinez, Konda Kumaraswami, Hellen Ishikawa-Ankerhold, Sarah Meister, Elisabeth Deindl

**Affiliations:** 1 Walter-Brendel-Centre of Experimental Medicine, University Hospital, LMU Munich, 80539 Munich, Germany; Manuel.Lasch@med.uni-muenchen.de (M.L.); a_caballeromartinez@hotmail.com (A.C.M.); Kumaraswami.Konda@med.uni-muenchen.de (K.K.); Hellen.Ishikawa-Ankerhold@med.uni-muenchen.de (H.I.-A.); 2 Department of Otorhinolaryngology, Head and Neck Surgery, University Hospital, LMU Munich, 80539 Munich, Germany; 3 Department of Internal Medicine I, Faculty of Medicine, University Hospital, LMU Munich, 80539 Munich, Germany; 4 Department of Obstetrics and Gynaecology, University Hospital, LMU Munich, 80539 Munich, Germany; Sarah.Meister@med.uni-muenchen.de

**Keywords:** arteriogenesis, collateral artery growth, SMC proliferation, potassium channel, K_V_1.3, K_Ca_3.1, FGFR-1, Egr-1, PDFG-R, αSM-actin

## Abstract

Collateral artery growth (arteriogenesis) involves the proliferation of vascular endothelial cells (ECs) and smooth muscle cells (SMCs). Whereas the proliferation of ECs is directly related to shear stress, the driving force for arteriogenesis, little is known about the mechanisms of SMC proliferation. Here we investigated the functional relevance of the potassium channels K_V_1.3 and K_Ca_3.1 for SMC proliferation in arteriogenesis. Employing a murine hindlimb model of arteriogenesis, we found that blocking K_V_1.3 with PAP-1 or K_Ca_3.1. with TRAM-34, both interfered with reperfusion recovery after femoral artery ligation as shown by Laser-Doppler Imaging. However, only treatment with PAP-1 resulted in a reduced SMC proliferation. qRT-PCR results revealed an impaired downregulation of α smooth muscle-actin (αSM-actin) and a repressed expression of fibroblast growth factor receptor 1 (*Fgfr1*) and platelet derived growth factor receptor b (*Pdgfrb*) in growing collaterals in vivo and in primary murine arterial SMCs in vitro under K_V_1.3. blockade, but not when K_Ca_3.1 was blocked. Moreover, treatment with PAP-1 impaired the mRNA expression of the cell cycle regulator early growth response-1 (*Egr1*) in vivo and in vitro. Together, these data indicate that K_V_1.3 but not K_Ca_3.1 contributes to SMC proliferation in arteriogenesis.

## 1. Introduction

Arteriogenesis, which is defined as the growth of pre-existing arteriolar connections into functional arteries compensating for the loss of an artery due to occlusion [1], particularly involves the proliferation of vascular endothelial cells (ECs) and smooth muscle cells (SMCs). The driving force for arteriogenesis is increased fluid shear stress [2,3]. This mechanical force, which can be sensed directly by ECs, has recently been shown to be linked to local activation of collateral ECs. By mediating the release of extracellular RNA (eRNA) from ECs, eRNA promotes the binding of vascular endothelial growth factor A (VEGFA) to VEGF receptor 2 (VEGFR2) [4], thereby promoting local vascular EC proliferation as well as activation of a mechanosensory complex, consisting of VEGFR2, platelet endothelial cell adhesion molecule 1 (PECAM-1), and vascular endothelial cell cadherin (VE-cadherin) [5], triggering collateral artery growth.

SMCs, located at the abluminal side, are not able to sense shear stress, and relatively little is known about the mechanisms triggering SMC proliferation in arteriogenesis. Growth factors, such as fibroblast growth factor 2 (FGF-2) and platelet-derived growth factor BB (PDGF-BB) have been shown to be important for SMC proliferation in arteriogenesis [6,7] (and own unpublished results of M. Lasch). This might in particular be related to their function to induce the expression of early growth response 1 (*Egr1*) [8], a transcriptional regulator, which has been shown to control cell cycle progression in arteriogenesis [9]. Interestingly, it has been demonstrated that the receptor for FGF-2, namely FGFR-1, which is expressed on collateral SMCs but not on ECs, is increased expressed only during a short time frame after induction of arteriogenesis [10]. These data indicate that the point in time of FGF-2 application is critical and might explain the outcome of clinical studies in which FGF-2 treatment showed limited effects in patients with vascular occlusive diseases [11,12]. 

The proliferation of vascular SMCs in arteriogenesis is characterized by the transition from the contractile to the synthetic (proliferating) SMC type. This process is associated with reduced mRNA and protein levels of contractile genes such as α-smooth muscle actin (αSM-actin) and paralleled by an increased expression of *Egr1*. [9,13,14]. The opposed gene expression of contractile genes and *Egr1*, which is tightly regulated by the transcriptional co-activators myocardin and myocardin-related transcription factors (MRTFs) and the ternary complex factor ETS like protein Elk-1, which compete for the binding to the transcription factor serum response factor (SRF) [15,16,17], has been demonstrated for the process of arteriogenesis by our group [9]. 

The potassium channels K_V_1.3 and K_Ca_3.1 have been shown to be involved in cell cycle regulation by activating intracellular signaling pathways [18,19], and to play a role in modulating vascular SMC proliferation [20,21]. Since the mechanisms triggering vascular SMC proliferation in arteriogenesis are still not very well described, we decided to investigate the functional contribution of K_V_1.3 and K_Ca_3.1 to the proliferation of vascular SMCs in collateral artery growth. The mode of action of these potassium channels in terms of proliferation is still under debate and several mechanisms, either ion flux dependent or independent, have been proposed [18,19]. Both potassium channels, K_V_1.3 and K_Ca_3.1, have been demonstrated to be upregulated in proliferating SMCs, whereby their specific blockade interfered with cell cycle progression. Increased expression levels of K_V_1.3 have been detected in vitro in proliferating SMCs isolated from murine femoral arteries or from human donors [21,22] as well as under pathological situations such as neointima hyperplasia in vivo [22,23]. Moreover, it has been shown that blockade of K_V_1.3 with selective blockers such as 5-(4-phenoxybutoxy)psoralen (PAP-1) inhibited migration and proliferation of SMCs in vitro [22,23] and in vivo [23,24]. Similar to K_V_1.3, K_Ca_3.1 has been found to be upregulated upon stimulation with PDGF-BB in proliferating SMCs in vitro [20] and in models of hyperplasia in vivo [25,26]. Blocking K_Ca_3.1 with the selective blocker TRAM-34 in contrast interfered with SMC proliferation both, in vitro and in vivo [20,25,27,28]. Besides its effect on SMC proliferation, K_Ca_3.1 plays a major role in endothelium-derived hyperpolarizing factor (EDHF)-mediated vasodilation as shown in K_Ca_3.1 deficient mice [29]. 

In the present study we investigated the relevance of the potassium channels K_V_1.3 and K_Ca_3.1 for SMC proliferation in growing collateral arteries by performing blocking studies employing the selective channel blockers PAP-1 and TRAM-34, respectively. From our results we conclude that K_V_1.3 contributes to SMC proliferation in arteriogenesis, whereas K_Ca_3.1 is more likely to be involved in vasodilation.

## 2. Materials and Methods

### 2.1. Animal Protocol and Treatments

**Figure 1:**
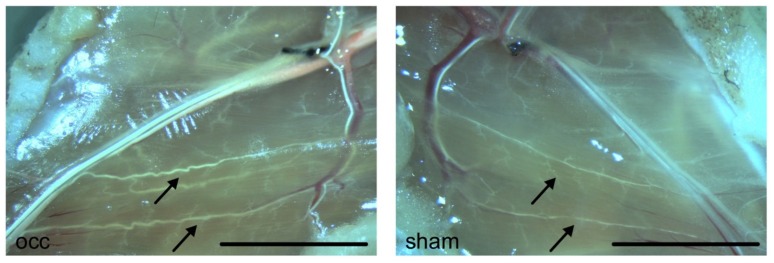
Photographs of superficial collateral arteries in mouse adductor muscles. Photographs were taken 7 days after induction of arteriogenesis by femoral artery ligation (left picture) or sham operation (right picture). Mice were perfused with latex to better visualize collateral arteries. Pre-existing collaterals appear very fine and straight (arrows, right picture). Seven days after induction of arteriogenesis, grown collateral arteries show a typical corkscrew formation with increased vascular caliber size (arrows, left picture). Scale bar 5 mm

Male C57BL6/J mice, purchased from Charles River, were housed in cages and kept under 12 h day/night cycle with food and water ad libitum. All experiments were approved by the Bavarian Animal Care and Use Committee (ethical approval code ROB-55.2-1-54-2532-73-12 and ROB-55.2Vet-2532.Vet_02-17-99) and carried out according to the guidelines of the German law for protection of animal life. Mice at the age of 6 to 10 weeks were anesthetized with a combination of 0.5 mg/kg medetomidine (Pfister Pharma), 5 mg/kg midazolam (Ratiopharm GmbH), and 0.05 mg/kg fentanyl (CuraMED Pharma). Arteriogenesis was induced by right femoral artery ligation (FAL, occlusion (occ)), whereas the left femoral artery was sham operated (Figure 1) as previously described in [30].

To block potassium channels, mice were treated either with the selective K_V_1.3 channel blocker (5-(4-phenoxybutoxy)psoralen (PAP-1, 40 mg/kg/d, intraperitoneally (i.p), Sigma-Aldrich) [31], or the selective K_Ca_3.1 channel blocker TRAM-34 (120 mg/kg/d, i.p., Alomone Labs) [27], dissolved in peanut oil, at doses previously described [27,31]. The treatments started 4 h before the surgical procedure. Moreover, to uphold constant blood levels of the blockers, mice received two doses per day, one in the morning and one in the afternoon. When mice were treated with BrdU (Sigma-Aldrich), they received a single dose (1.25 mg/d dissolved in phosphate buffered saline (PBS), i.p.) starting directly after the surgical procedure.

### 2.2. Laser Doppler Perfusion Measurements and Tissue Sampling

The laser Doppler perfusion measurements were performed as described in [4]. In brief, hindlimb perfusion was measured using the laser Doppler imaging technique (Moor LDI2-IR, LDI 5061 and Moor Software 3.01, Moor Instruments) under temperature-controlled conditions (37 °C), and perfusion was calculated by right to left (occlusion (occ) to sham) flux ratios.

Prior to tissue sampling for (immuno-) histology, mice were perfused with an adenosine buffer (1% adenosine, 5% bovine serum albumin (BSA), both from Sigma-Aldrich dissolved in PBS, PAN Biotech, pH 7.4) for maximal vasodilation followed by perfusion with 3% paraformaldehyde (PFA, Merck) dissolved in PBS, pH 7.4, for cryoconservation, or 4% PFA for paraffin embedding [2]. For qRT-PCR analyses, mice were perfused with latex flexible compound (Chicago Latex) to visualize superficial collateral arteries (see also Figure 1) for dissection. After isolation, superficial collateral arteries were snap frozen on dry ice and stored at −80 °C until further investigations [9].

### 2.3. Cell Culture

Mouse primary artery smooth muscle cells (catalog number C57-6081, CellBiologics) were cultured in a SMC growth medium (SMCGM, CellBiologics) containing insulin and the growth factors fibroblast growth factor 2 (FGF-2) and epidermal growth factor (EGF) together with 20% fetal calf serum (FCS, PAN). For serum starvation, cells were cultured in Dulbecco’s modified Eagle´s medium (DMEM, Thermo Fisher Scientific) with 1% FCS for 24 h. Thereafter, negative controls were stimulated with medium containing 2% FCS, positive controls with 10% FCS.

### 2.4. Histology, Immunohistology, Proliferation Assay, and Immunocytochemistry

Giemsa staining on paraffin fixed tissue samples was performed according to standard procedures, and slices were analyzed using an Axioskop 40 microscope (Carl Zeiss AG). BrdU staining of paraffin fixed tissue sections was performed with a BrdU detection kit (BD Pharmingen) according to the manufacturer´s procedure using the same microscope for evaluating the tissue sections.

To investigate the proliferation of mouse primary artery SMCs, a BrdU proliferation assay kit (Roche) was used according to the manufacturer´s instructions. In brief, mouse primary artery SMCs were seeded in a 96-well plate overnight, and after serum starvation in DMEM containing 1% FCS for 24 h, the mouse primary artery SMCs were cultured in DMEM with 10% FCS and treated with or without PAP-1 or TRAM-34, respectively, together with 10 mM BrdU. Cell proliferation was assessed by colorimetry with an Infinite F200 ELISA reader (TECAN).

For immunofluorescence staining, cryofixed tissue sections (10 μm) were stained with a rabbit anti-K_V_1.3 (catalog number APC-101) or a rabbit anti-K_Ca_3.1 (catalog number APC-064) antibody (both from Alomone Labs) followed by a goat anti-rabbit IgG Alexa fluor 488-conjugated antibody (catalog number 711-545-153, Jackson ImmunoResearch) together with a Cy3-conjugated mouse anti-αSM-actin antibody (catalog number C6198, Sigma-Aldrich) and an Alexa fluor 647-conjugated rat anti-CD31 antibody (catalog number 102515, BioLegend), followed by DAPI counter staining (catalog number 62248, Thermo Fisher Scientific). Images were taken with an Axio Imager 2 fluorescence microscope equipped with and an Axion ICc 5 camera and Axiovert software (Carl Zeiss) or using a LSM 880 confocal laser scanning microscope equipped with an Airycan module (Carl Zeiss) with ZEN black software for imaging acquisition. Imaging analysis of K_V_1.3 or K_Ca_3.1 expression in αSM-actin positive SMCs or CD31 positive ECs was performed using the ZEN blue software. For the colocalization anaylsis, the ZEN colocalization tool was used (Carl Zeiss AG). The three-dimensional (3D) projection surface reconstruction of the images where done by using the Imaris software (Bitplane).

### 2.5. RNA Isolation, cDNA Synthesis, and qRT-PCR

The total RNA was isolated from the mouse primary artery SMCs or collateral arteries with Trizol (Life Technologies) and the residual DNA was removed by digestion with RQ1 RNase-Free DNase (Promega). Thereafter, RNA was purified with RNeasy MinElute columns (Qiagen) and reverse transcribed to cDNA using the QuantiTect^®^ Reverse Transcription Kit (Qiagen) according to the manufacturer´s procedure. The qRT-PCR was performed as previously described [32] using the Power SYBR Green Kit (Life Technologies) and a StepOnePlus cycler (Life Technologies) and the following primers: 18S rRNA forward 5′-GGACAGGATTGACAGATTGATAG-3′, reverse 5′-CTCGTTCGTTATCGGAATTAAC-3′, αSM-actin forward 5′-GAGCATCCGACACTGCTG-3′, reverse 5′-GTACGTCCAGAGGCATAG-3′, fibroblast growth factor receptor-1 (*Fgfr1*) forward 5′-CTTGCCGTATGTCCAGATCC-3′, reverse 5′-TCCGTAGATGAAGCACCTCC-3′, platelet derived growth factor b (*Pdgfrb*) forward 5′-AGGACAACCGTACCTTGGGTGACT-3′, reverse 5′-CAGTTCTGACACGTACCGGGTCTC-3′, early growth response 1 (*Egr1*) forward 5′-CGAACAACCCTATGAGCACCTG-3′, and reverse 5′-CAGAGGAAGACGATGAAGCAGC-3′. To control specific amplification, melt curve analyses and agarose gels were performed. Data were analyzed using the ΔΔCt method [33] and results were normalized to the expression level of the 18S rRNA.

### 2.6. Statistical Analyses

Statistical analyses were performed using the GraphPad software PRISM6. All data are stated as means ± SEM. Results were tested for normality and statistical analyses were performed as specified in the figure legends. Results were considered to be statistically significant at *p* ≤ 0.05.

## 3. Results

### 3.1. K_V_1.3 and K_Ca_3.1 are Localized in Collateral Arteries

**Figure 2:**
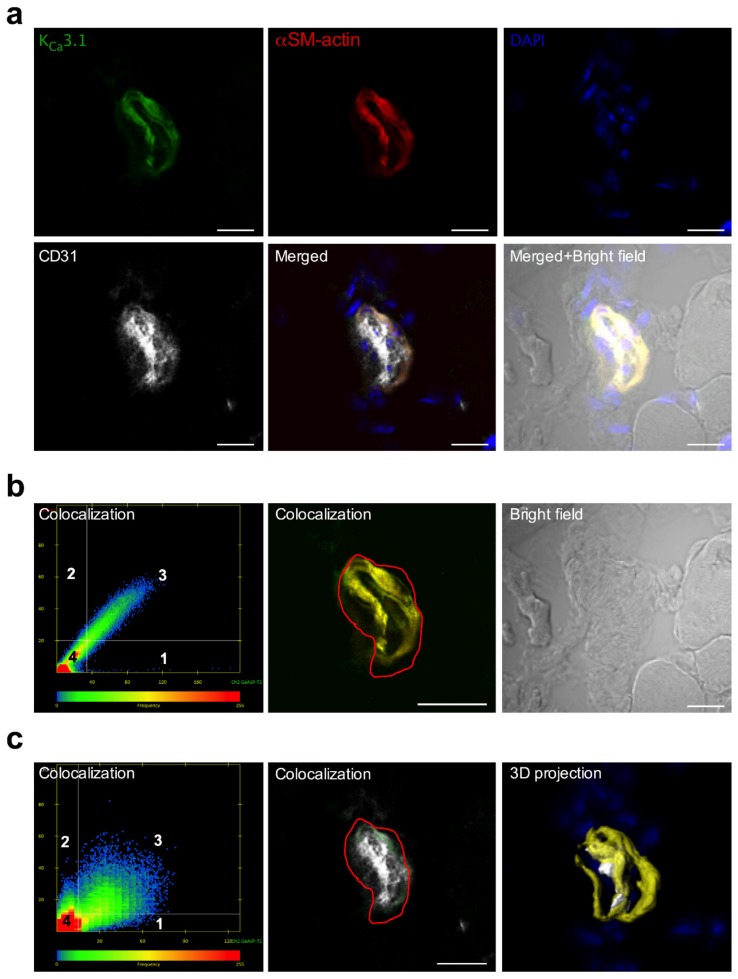
Localization of K_Ca_3.1 in ECs and SMCs of murine collateral arteries. (**a**) Representative confocal immunofluorescence images of transversal sections of collateral arteries isolated 3 h after induction of arteriogenesis. Tissue sections were stained with an antibody against K_Ca_3.1 (green), together with the SMC marker αSM-actin (red), the EC marker CD31 (grey), and DAPI (blue); (**b**,**c**) Scatterplots showing the colocalization analysis, (left lower panel) represents pixels that have low intensity levels in both channels, green and red (**b**), or green and gray (**c**). Quadrant 4 (lower left bottom) represents pixels that are referred to as background and are not taken into consideration for colocalization analysis. Quadrant 1 represents pixels that have high green intensities and low red intensities and Quadrant 2 represents pixels that have high red intensities and low green intensities. Quadrant 3 represents pixels with high intensity levels in both green and red (b) or green and gray (**c**). These pixels are considered to be colocalized. Bright field image is also displayed. (**c**) 3D projection surface rendering is showing the localization of the K_Ca_3.1 with the labelling CD 31 and αSM-actin display on the panel (**c**) right lower position. Scale bar 20 µm.

**Figure 3:**
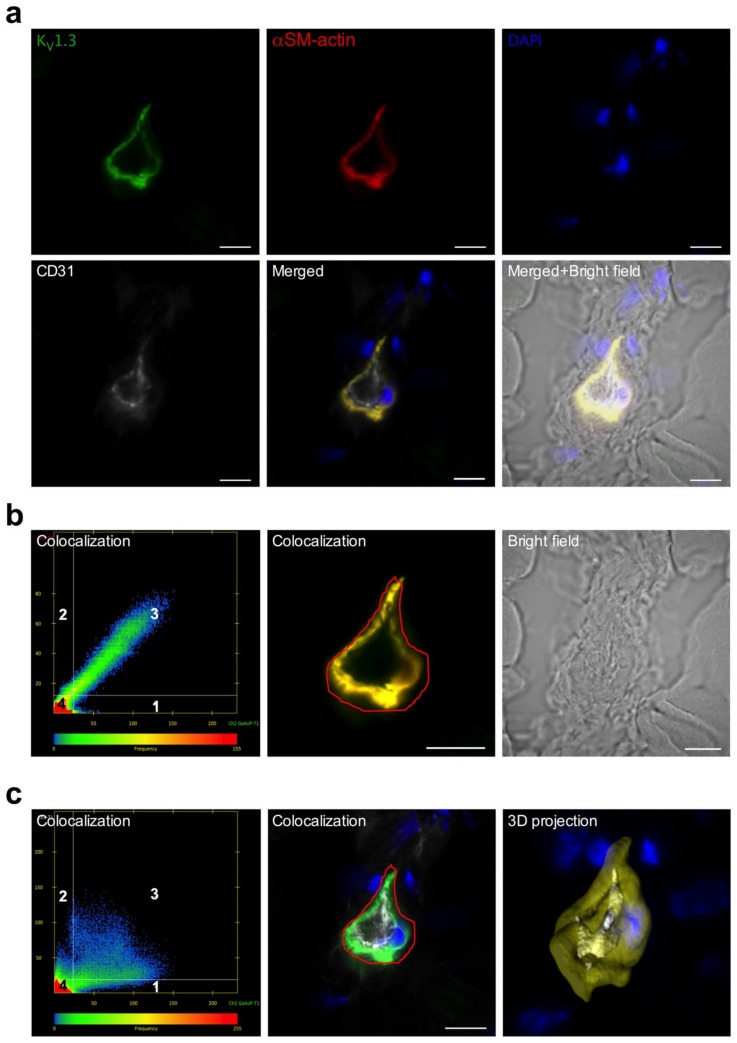
Localization of K_V_1.3 in ECs and SMCs of murine collateral arteries. (**a**) Representative confocal immunofluorescence images of transversal sections of collateral arteries isolated 3 h after induction of arteriogenesis. Tissue sections were stained with an antibody against K_V_1.3 (green), together with the SMC marker αSM-actin (red), the EC marker CD31 (grey), and DAPI (blue); (**b**,**c**) Scatterplots showing the colocalization analysis. Quadrant 4 (left lower left panel) represents pixels that have low intensity levels in both channels, green and red (**b**) or green and grey (**c**), and these pixels are referred to as background and are not taken into consideration for colocalization analysis. Quadrant 1 represent pixels that have high green intensities and low red intensities and Quadrant 2 represents pixels that have high red intensities and low green intensities. Quadrant 3 represents pixels with high intensity levels in both green and red in (**b**) and green and grey in (**c**). These pixels are considered to be colocalized. Scale bar 20 µm. Bright field image is also displayed. (**c**) 3D projection surface rendering is showing the localization of the K_V_1.3 with the labelling CD 31 and αSM-actin on the panel (**c**) right lower position.

Employing a murine hindlimb model of arteriogenesis, we investigated whether the potassium channel K_V_1.3 or K_Ca_3.1, respectively, were expressed in adductor collateral arteries. Immunofluorescence imaging revealed that K_V_1.3 and K_Ca_3.1 labelling strongly colocalized with αSM-actin, a marker for SMCs, but weakly with CD 31, which is a marker for ECs (Figure 2 and Figure 3).

### 3.2. Blockade of K_V_1.3 But Not of K_Ca_3.1 Impaired Arteriogenesis by Inhibiting Collateral SMC Proliferation

**Figure 4:**
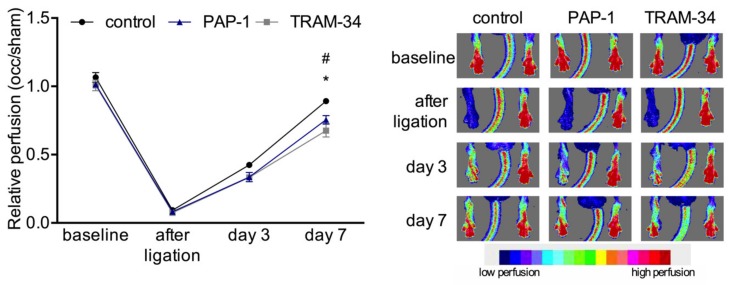
Laser Doppler perfusion measurements. Line plot (left panel) along with corresponding flux images (right panel) of laser Doppler perfusion measurements. Mice were treated with solvent (control), PAP-1, or TRAM-34, respectively, and the perfusion was calculated by right to left (occlusion (occ) to sham) ratio before, immediately after, and at day 3 and 7 after the surgical procedure (left panel). Data are means ± SEM, *n* = 6 per group. * *p* < 0.05 (PAP-1 vs. control) and # *p* < 0.05 (TRAM-34 vs. control) from two-way ANOVA with Bonferroni’s multiple comparison test. The right panel shows representative flux images of murine paws with the tail in the center. Cold colors (blue, green) indicate low perfusion, whereas warm colors (yellow, red) indicate high perfusion (see scale).

To investigate the functional relevance of the potassium channels for arteriogenesis, the K_V_1.3 channel was blocked with PAP-1, and the K_Ca_3.1 channel with TRAM-34. The laser Doppler perfusion measurements revealed that both treatments significantly interfered with reperfusion recovery after femoral artery ligation (Figure 4).

**Figure 5:**
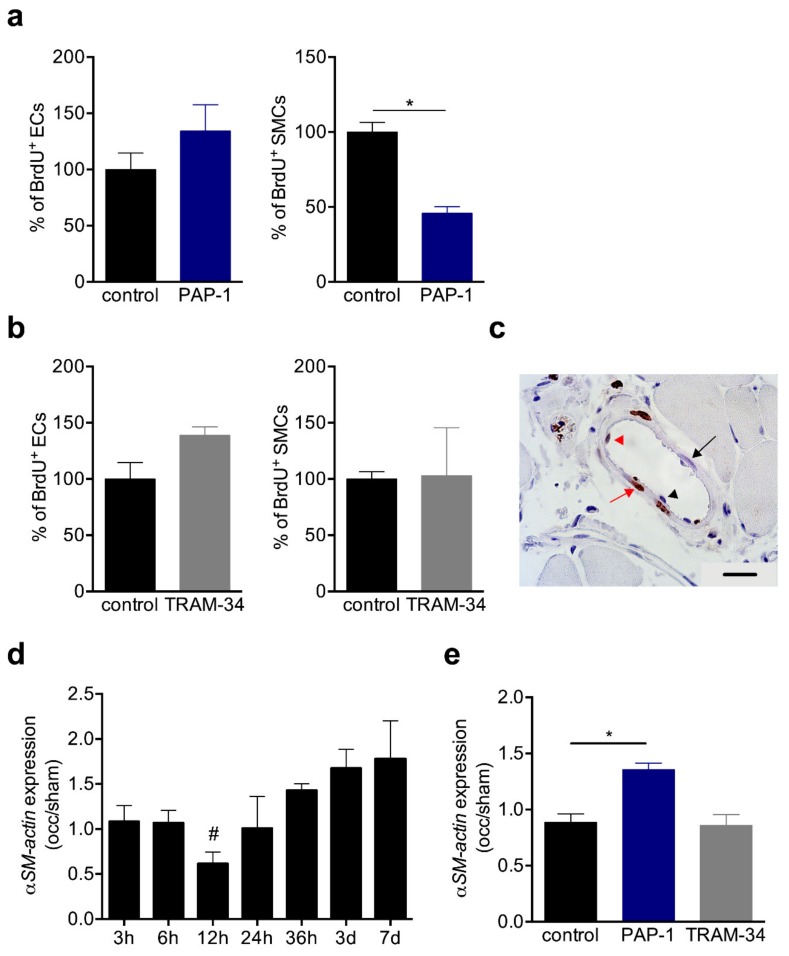
BrdU incorporation and αSM-actin expression in collaterals. (**a**,**b**) Bar graphs represent the results of quantitative analyses of BrdU^+^ ECs (left panels) and SMCs (right panels) in solvent (control), (**a**) PAP-1 or (**b**) TRAM-34-treated mice at day 7 after induction of arteriogenesis. Data are means ± SEM, *n* = 3 mice per group. * *p* < 0.05 from unpaired student´s t-test. The numbers of BrdU^+^ cells in control collaterals were defined as 100%; (**c**) Representative picture of a BrdU stained collateral at day 7 after induction of arteriogenesis. Scale bar 20 µm; (**d**,**e**) The bar graphs represent the expression levels of αSM-actin (occlusion/sham (occ/sham)) in collateral arteries (**d**) at different time points after induction of arteriogenesis or (**e**) at 12 h after induction of arteriogenesis in control, PAP-1, or TRAM-34 treated mice. The qRT-PCR results were normalized to the expression level of the 18SrRNA. Data are means ± SEM, n > 3 per group. * *p* < 0.05 from unpaired student’s t-test and refers in (**d**) to occ vs. sham.

To quantify the effects of channel blockade on vascular cell proliferation, we performed immunohistochemical analyses of the proliferation marker BrdU in transversal sections of collateral arteries at day 7 after induction of arteriogenesis. The results showed that both treatment with the K_V_1.3 blocker PAP-1 and with K_Ca_3.1 blocker TRAM-34 did not interfered with EC proliferation in growing collaterals. However, the PAP-1 treatment significantly reduced SMC proliferation, an effect that was not observed when mice were treated with TRAM-34 (Figure 5a–c).

During the transition from the synthetic to the proliferative phase, the mRNA expression level of αSM-actin has been shown to be downregulated 12h after induction of arteriogenesis [9], and confirmed in the present study by qRT-PCR analyses (Figure 5d). Interestingly, in TRAM-34 treated mice, the expression level of αSM-actin was comparable to that of the control mice at 12 h after induction of arteriogenesis, however, it was significantly increased in PAP-1 treated mice at the same point in time (Figure 5e).

### 3.3. K_V_1.3 and K_Ca_3.1 Blockade Inhibits Mouse Primary Artery SMCs Proliferation In Vitro

**Figure 6:**
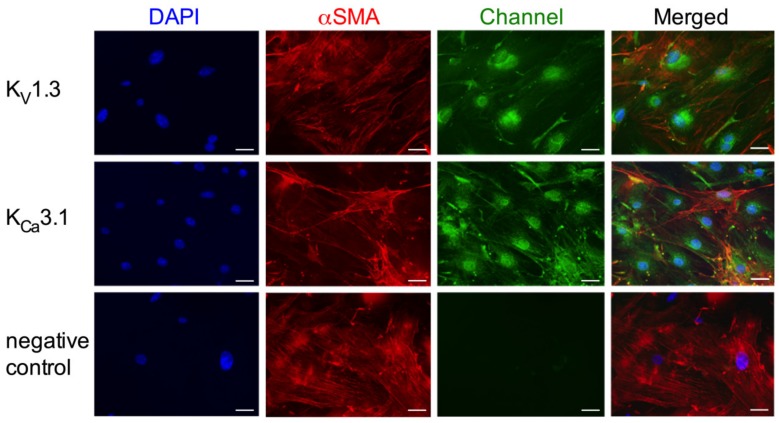
Immunocytological analyses on K_V_1.3 and K_Ca_3.1 localization in mouse primary artery SMCs. Cells were stained with antibodies against the K_V_1.3 (upper panels, green) or the K_Ca_3.1 channel (middle panels, green) together with an antibody against the SMC marker αSM-actin (red) and counterstained with DAPI (blue) to show the nuclei. For negative control (lower panels) the primary antibody was omitted. Scale bar 40 µm.

To gain further insights into the role of the potassium channels on SMC proliferation, we performed in vitro investigations on mouse primary artery SMCs. Immunocytological analyses showed that K_V_1.3, as well as K_Ca_3.1, are localized perinuclear in mouse primary artery SMCs. Somehow, weaker signals were seen in the cytoplasm and at the cytoplasmic membrane (Figure 6).

**Figure 7:**
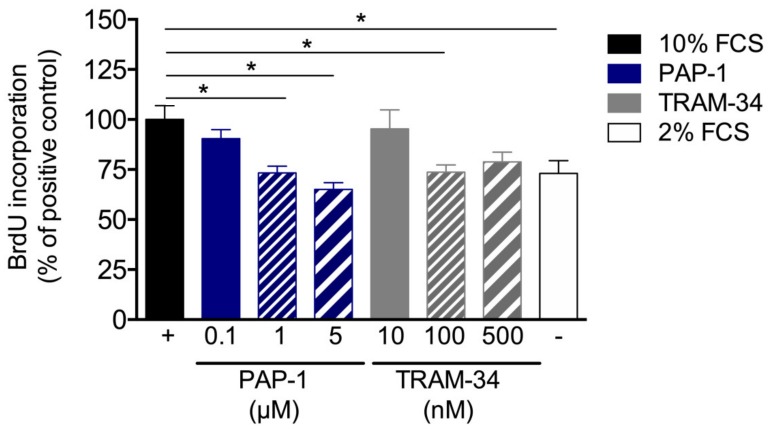
Proliferation assay of mouse primary artery SMCs. Mouse primary artery SMCs were cultured with 10% FCS with or without treatment of different concentrations of the K_V_1.3 blocker PAP-1 or the K_Ca_3.1 blocker TRAM-34. Cell proliferation was investigated by means of BrdU incorporation. Values are expressed as percentages of the positive control (+), i.e., mouse primary artery SMCs stimulated with 10% FCS. For the negative control (–), mouse primary artery SMCs cultured with 2% FCS. Data are means ± SEM, *n* > 6 per group. * *p* < 0.05 from one-way ANOVA with Bonferroni’s multiple comparison test.

To analyze the effects of K_V_1.3 and K_Ca_3.1 blockade on SMC proliferation in in vitro mouse, primary artery SMCs were treated with different concentrations of PAP-1 (0.1, 1, and 5 μM) or TRAM-34 (10, 100, and 500 μM), respectively. Interestingly, in in vitro, both PAP-1 and TRAM-34 treatments interfered with SMC proliferation, as shown by the BrdU incorporation assay (Figure 7).

### 3.4. K_V_1.3 Blockade Repressed the Expression of FGFR-1, PDGFR-ß, and Egr1 in Mouse Primary Artery SMCs In Vitro and During Arteriogenesis In Vivo

**Figure 8:**
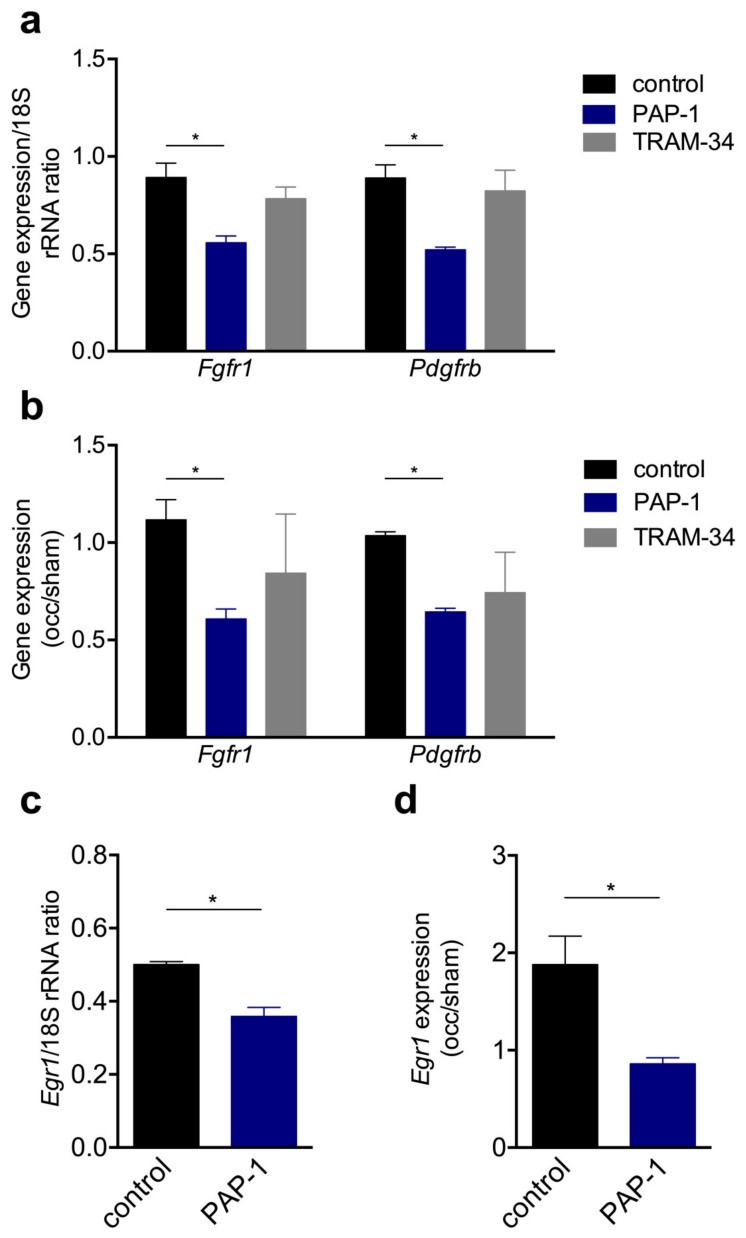
The qRT-PCR results of the expression levels of *Fgfr1*, *Pdgfrb,* and *Egr1* in vitro and during arteriogenesis in vivo. (**a**,**c**) Bar graphs represent the mRNA expression levels of Fgfr1, Pdgfrb, or Egr1 in vitro and (**b**,**d**) in vivo. In vitro mouse primary artery SMCs were cultured without (control) or with 1 μM PAP-1 or 100 nM TRAM-34, respectively. In vivo the expression level of *Fgfr1*, *Pdgfrb,* and *Egr1* were investigated 12 h after induction of arteriogenesis in collateral arteries and are expressed as occlusion (occ) to sham ratio. All qRT-PCR results were normalized to the expression level of the corresponding 18S rRNA. Data are means ± SEM, *n* = 3 per group. * *p* < 0.05 from one-way ANOVA with Bonferroni’s multiple comparison test.

Receptor tyrosine kinases such as FGFR-1 and PDGFR-ß are well described for their relevance in SMC proliferation. Our qRT-PCR results on the expression level of *Fgfr1* and *Pdgfrb* provided evidence that treatment of mouse primary artery SMCs with the K_V_1.3 channel blocker PAP-1 significantly interfered with the expression of both growth factor receptors, whereas the treatment with the K_Ca_3.1 channel blocker TRAM-34 showed no significant influence (Figure 8a). Moreover, in collateral arteries 12 h after induction of arteriogenesis, a significant downregulation was evident for both *Fgfr1* and *Pdgfrb* when K_V_1.3 was blocked with PAP-1, while treatment with the K_Ca_3.1 blocker TRAM-34 showed no significant effect (Figure 8b). To further investigate the relevance of the K_V_1.3 potassium channel for SMC proliferation in vitro and during arteriogenesis in vivo, qRT-PCR analyses were performed on the cell cycle regulator Egr1. Our results evidenced that blocking K_V_1.3 with PAP-1 in vitro, as well during arteriogenesis in vivo, significantly interfered with the mRNA expression of *Egr1* (Figure 8c,d).

## 4. Discussion

The process of arteriogenesis mainly involves the proliferation of ECs and SMCs. Whereas the mechanisms relevant for EC proliferation are relatively well defined, little is known about the mechanism involved in SMC proliferation. Using a murine hindlimb model of arteriogenesis, here, we report that the voltage-gated potassium channel K_V_1.3, but not the Ca^2+^-gated potassium channel K_Ca_3.1, is of importance for SMC proliferation in collateral arteries. Selectively blocking K_V_1.3 with PAP-1 resulted in a reduced perfusion recovery (Figure 4), which was associated with reduced numbers of proliferating SMCs (Figure 5). More in-depth in vivo and in vitro studies demonstrated a role for K_V_1.3 in the expression of the tyrosine kinase receptors *Fgfr1* and *Pdgfrb,* as well as of the transcriptional regulator *Egr1* (Figure 8), all relevant for proper SMC proliferation in arteriogenesis.

Elevated shear stress is the driving force for arteriogenesis [2,3]. This mechanical stress can be sensed by ECs but not by SMCs. The mechanisms how this mechanical force is translated into biochemical signals resulting in endothelial proliferation have been described in [4]. However, little is known about the mechanisms triggering SMC proliferation in arteriogenesis. To address this point, we decided to study the relevance of the potassium channels K_v_1.3 and K_Ca_3.1, which have been shown to play a role in smooth muscle proliferation in other experimental settings and processes [20,21]. Our immunohistological investigations demonstrated that both K_v_1.3 and K_Ca_3.1 are mainly localized in SMCs of collateral arteries of murine hindlimbs (Figure 2 and Figure 3).

To investigate the relevance of K_V_1.3 for arteriogenesis, we performed blocking studies employing PAP-1, described as a selective K_V_1.3 blocker [31]. The laser Doppler perfusion measurements evidenced a significant reduction in perfusion recovery when mice were treated with PAP-1 (Figure 4). Moreover, our histological results showed a significant reduction of proliferating SMCs but not ECs in growing collateral arteries (Figure 5).

Proliferating SMCs are characterized by a reduced expression of the contractile marker αSM-actin *a* [34], which has been demonstrated by our group for the process of arteriogenesis [9] and confirmed in the present study (Figure 5). Blocking K_V_1.3 during collateral artery growth, however, interfered with the downregulation of αSM-actin (Figure 5). To investigate whether the reduced proliferation rate of SMCs in arteriogenesis was directly related to K_V_1.3 blockade in SMCs, but not in other cells such as leukocytes, which also play an important role in arteriogenesis [18,35,36], we analyzed the proliferative behavior of primary murine SMCs under K_V_1.3 blockade in vitro. Our results revealed a correlation between the concentration of PAP-1 in culture medium and the inhibition of mouse primary artery SMC proliferation (Figure 7), attributing K_V_1.3 with a role in SMC proliferation. Together, our data suggest a direct correlation between the blockade of the potassium channel K_V_1.3 and the inhibition of SMC proliferation during the process of arteriogenesis. Our data are in line with results from Cidad et al., who demonstrated an inhibition of femoral artery SMC proliferation when K_v_1.3 was blocked pharmacologically with PAP-1 or with Margatoxin or when K_v_1.3 was knocked down by siRNA treatment [22].

Previous results have shown that SMC proliferation in arteriogenesis is dependent on the activation of FGFR-1. Already in 2003, we had demonstrated that FGFR-1, which was expressed in SMCs but not in ECs, was upregulated in the early phase of arteriogenesis, i.e., within the first 24 h after induction of collateral artery growth by femoral artery ligation, and that blocking this tyrosine kinase receptor with polyanetholsulfonic acid (PAS) interfered with the process of arteriogenesis [10]. A parallel study showed that a combined treatment of rodents with the cognate ligand of FGFR-1, namely FGF-2, and the cognate ligand of PDGFR-β, namely PDGF-BB, significantly promoted the process of arteriogenesis [6]. In that study, an upregulation of PDGF receptors by FGF-2 was suggested and was later confirmed by Zhang et al. [37]. PDGF-BB has been described as a potent inducer of the synthetic phenotype of a SMC and has been shown to act synergistically with FGF-2 to induce the downregulation of contractile genes such as αSM-actin during vascular SMC proliferation [38]. In particular, it has been demonstrated that PDGF-BB activates FGFR-1 via engaging PDGFR-β, thereby mediating the downregulation of αSM-actin and smooth muscle 22α (SM22-α) expression. The PDGFR-β/PDGF-BB and FGFR-1/FGF-2 signaling pathways have also been effectively described to promote the upregulation of the transcription factor Egr1 [8], the expression of which was regulated in opposition to that of contractile genes, and which we have found to mediate cell cycle progression in arteriogenesis [9]. In the present study, we found that treatment of mice with the K_v_1.3 blocker PAP-1 during the process of arteriogenesis resulted in a downregulation of *Fgfr1*, *Pdgfrb*, and *Egr1* (Figure 8), whereas αSM-actin was upregulated (Figure 5). Accordingly, all genes are regulated in the opposite way as described for proper arteriogenesis [9,10]. Although one could speculate that the impaired expression of *Fgfr1* and/or *Pdgfrb* could be responsible for the impaired expression of their downstream genes, i.e., *Egr1* and αSM-actin, this is somehow unlikely as all genes show a hampered expression at the same point of time. Therefore, we wondered if another factor could be involved in K_v_1.3 mediated gene expression. Interestingly, in silico analyses (data not shown) revealed several binding sites for the transcription factor specificity protein 1 (Sp1) in the promoter regions of *Fgfr1*, *Pdgfrb*, and *Egr1*. However, whether Sp1 is indeed involved in potassium channel K_v_1.3 mediated gene expression remains to be determined by further studies. Our data indicate that K_v_1.3 plays a major role in SMC proliferation, especially in the process of arteriogenesis, by influencing signal transduction cascades associated with the expression of the growth factor receptors *Fgfr1* and *Pdgfrb* and their downstream genes being involved in phenotype switch and cell cycle regulation.

In contrast to our findings regarding PAP-1 administration, treatment of mice with the K_Ca_3.1 selective blocker TRAM-34 did not influence vascular SMC proliferation or differential gene expression in growing collaterals in vivo (Figure 5 and Figure 8). The laser Doppler perfusion measurements, however, evidenced a reduced perfusion recovery after femoral artery ligation (Figure 4). Interestingly, K_Ca_3.1 has been shown to be upregulated by fluid shear stress [39], the driving force for arteriogenesis [2,3]. Moreover, it has been demonstrated by blocking studies employing TRAM-34 in vivo, that K_Ca_3.1 plays a role in EC proliferation during angiogenesis (Grigic, Eichler 2005) and in SMC proliferation, e.g., during atherogenesis [27]. Our study, however, revealed that K_Ca_3.1 is not involved in EC or in SMC proliferation in collateral artery growth (Figure 5). Of course, one could argue that the dose of TRAM-34 used in the present study was not high enough to block K_Ca_3.1 in vivo, but identical dosages were shown to be effective in hampering vascular cell proliferation in a model of intima hyperplasia [25] and atherosclerotic lesions in mice [27]. Together, these data indicate that the mechanisms of SMC proliferation in the different pathophysiological situations are diverging. Indeed, it has been shown by Bi et al. in vitro [20] that K_Ca_3.1 mediated SMC proliferation blocked by TRAM-34 was not associated with any change in expression of *Pdgfrb*, supporting the data of the present investigations (Figure 8). As our laser Doppler perfusion measurements revealed a reduced perfusion recovery upon femoral artery ligation (Figure 4), which was not associated with a reduced collateral artery cell proliferation (Figure 5), we hypothesize that K_Ca_3.1 could overtake a function in EDHF-mediated collateral vasodilation, a well described function of this potassium channel [29,40]. However, further studies are necessary to prove this hypothesis. A similar effect on reduced perfusion recovery upon femoral artery ligation has been described for nitric oxide synthase 3 (NOS3)-deficient mice, also attributing nitric oxide a role in vasodilation during arteriogenesis [41]. In terms of K_Ca_3.1, it could be interesting to know that the Ca^2+^-channel transient receptor potential cation channel, subfamily V, member 4 (TRPV4) has previously been shown to play a role in arteriogenesis by promoting vascular cell proliferation [42]. TRPV4 is described as shear stress sensitive channel which plays an important role in the regulation of vascular tone by modulating intracellular Ca^2+^ levels [43]. However, TRPV4 has also been shown to promote collateral artery growth in several animal models [42,44,45]. It has been suggested that upon activation of this receptor, a first increase in intracellular Ca^2+^ levels could result in EDHF-mediated vasodilation, whilst a prolonged raise could activate transcription factors causing vascular cell proliferation [42,45]. It is tempting to speculate that K_Ca_3.1 is involved in this EDHF mediated vasodilation, but further studies are necessary to investigate this assumption.

## 5. Conclusions

From our investigations, we conclude that the potassium channel K_v_1.3, but not K_Ca_3.1, contributes to SMC proliferation in arteriogenesis by controlling the expression of growth factor receptors, as well as their downstream genes relevant for phenotype switch and cell cycle progression.
